# Improving antimicrobial prescribing for upper respiratory infections in the emergency department: Implementation of peer comparison with behavioral feedback

**DOI:** 10.1017/ash.2021.240

**Published:** 2021-12-23

**Authors:** George F. Jones, Valeria Fabre, Jeremiah Hinson, Scott Levin, Matthew Toerper, Jennifer Townsend, Sara E. Cosgrove, Mustapha Saheed, Eili Y. Klein

**Affiliations:** 1 Division of Infectious Diseases, Department of Medicine, Johns Hopkins University School of Medicine, Baltimore, Maryland; 2 Eastern Virginia Medical School, Norfolk, Virginia; 3 Department of Emergency Medicine, Johns Hopkins University School of Medicine, Baltimore, Maryland; 4 Division of Infectious Diseases, Greater Baltimore Medical Center, Towson, Maryland; 5 Center for Disease Dynamics, Economics & Policy, Washington DC

## Abstract

**Objective:**

To reduce inappropriate antibiotic prescribing for acute respiratory infections (ARIs) by employing peer comparison with behavioral feedback in the emergency department (ED).

**Design:**

A controlled before-and-after study.

**Setting:**

The study was conducted in 5 adult EDs at teaching and community hospitals in a health system.

**Patients:**

Adults presenting to the ED with a respiratory condition diagnosis code. Hospitalized patients and those with a diagnosis code for a non-respiratory condition for which antibiotics are or may be warranted were excluded.

**Interventions:**

After a baseline period from January 2016 to March 2018, 3 EDs implemented a feedback intervention with peer comparison between April 2018 and December 2019 for attending physicians. Also, 2 EDs in the health system served as controls. Using interrupted time series analysis, the inappropriate ARI prescribing rate was calculated as the proportion of antibiotic-inappropriate ARI encounters with a prescription. Prescribing rates were also evaluated for all ARIs. Attending physicians at intervention sites received biannual e-mails with their inappropriate prescribing rate and had access to a dashboard that was updated daily showing their performance relative to their peers.

**Results:**

Among 28,544 ARI encounters, the inappropriate prescribing rate remained stable at the control EDs between the 2 periods (23.0% and 23.8%). At the intervention sites, the inappropriate prescribing rate decreased significantly from 22.0% to 15.2%. Between periods, the overall ARI prescribing rate was 38.1% and 40.6% in the control group and 35.9% and 30.6% in the intervention group.

**Conclusions:**

Behavioral feedback with peer comparison can be implemented effectively in the ED to reduce inappropriate prescribing for ARIs.

Inappropriate prescribing of antibiotics exposes patients to unnecessary risks of adverse events, such as *Clostridioides difficile* infections, and contributes to the global antibiotic resistance crisis. Acute respiratory infections (ARIs) account for ∼10% of all ambulatory visits (including emergency department [ED] visits) in the United States.^1^ Despite evidence-based guidelines for antibiotic prescribing for ARIs, ∼50% of all outpatient antibiotic prescriptions for ARIs are considered unnecessary.^2^ EDs have a per-visit antibiotic prescribing rate that is nearly double that of office-based clinicians; however, because they typically see sicker patients, inappropriate prescribing in EDs is only slightly higher than at medical offices, though it remains a significant issue.^3^ Although ED clinicians understand the problems related to antibiotic resistance and inappropriate prescribing,^4,5^ there has not been significant change in practice.^2,6^

Several approaches to improve antibiotic use have been evaluated in different settings, including academic detailing, computerized clinical decision support, and financial incentives. Although these initiatives have largely been associated with reductions in antibiotic prescription rates, the improvements have generally been modest and lacked durability.^7–9^ In recent years, as electronic health records (EHRs) have become more common, alerts and reminders have been proposed as a mechanism to improve prescribing, but these can contribute to alarm fatigue and information overload, and they are often ignored.^10^ Clinician feedback, particularly when clinicians are compared with their peers, has shown effectiveness in reducing inappropriate antibiotic prescribing in ambulatory settings for patients presenting with ARIs.^7,9,11,12^ This approach has not been well studied in adult EDs, where inappropriate prescribing is known to be high.^13^ One study combined peer comparison with intensive face-to-face meetings and found a reduction in antimicrobial use for uncomplicated ARIs in the EDs, but this study did not evaluate feedback alone and included only willing participants.^14^ Given the potential of peer comparison interventions, we implemented a quality improvement project that aimed to reduce inappropriate prescribing for ARIs in the adult ED setting through an attending-physician feedback intervention targeting inappropriate antibiotic prescribing for ARIs.

## Methods

### Study design, setting, and population

We conducted a before-and-after, quasi-experimental initiative in the adult EDs at 5 hospitals in the Baltimore–Washington, DC, area (Table 1). These hospitals are all part of the Johns Hopkins Health System; they are diverse in type (ie, academic, teaching, and community hospitals); and they utilize a common EHR system (Epic, Verona, WI). The structure of hospital management includes 3 EDs at hospitals A, B and C (intervention group) that have a common department chair and share clinicians. The intervention was performed at these locations out of convenience. The other 2 hospitals D and E (control group) were not included in the intervention. The preintervention period was January 1, 2016, to March 31, 2018, and the postintervention period was April 1, 2018, to December 31, 2019.

**Table 1.** Characteristics of Participating Emergency Departments

Site	Location	Type of Hospital	No. of ED Encounters Annually	Treats Children
Hospital A	Baltimore, MD	Academic, urban	65,500	No
Hospital B	Baltimore, MD	Teaching, urban	50,000	No
Hospital C	Columbia, MD	Community, suburban	60,000	No
Hospital D	Bethesda, MD	Community, suburban	41,200	No
Hospital E	Washington, DC	Community, urban	40,800	Yes

Note. ED, Emergency department.

All encounters within the study period with patients aged ≥18 years that carried an *International Classification of Diseases, Tenth Revision* (ICD-10) code indicating a respiratory condition (Supplementary Tables 1 and 2) were included. Encounters that included an ICD-10 code indicating a non-respiratory condition that warrants or may warrant antibiotics (Supplementary Table 3) were excluded. Encounters were not excluded solely due to patients having an underlying condition such as cystic fibrosis, tracheostomy, or ventilator dependency. This quality improvement study was reviewed and approved by the Johns Hopkins Institutional Review Board.

### Developing lists of inclusion and exclusion diagnoses

Following the methods of the behavioral intervention implemented by Meeker et al,^7^ we developed a list of ICD-10 codes for upper respiratory system conditions for which the prescription of antibiotics is considered inappropriate (Supplementary Table 1). The list was reviewed by several ED and infectious disease clinicians practicing in the Johns Hopkins Health System. Because patients can have multiple diagnoses associated with an encounter, we also compiled a list of diagnoses for which the provision of antibiotics are or may be required. These diagnoses were further split into respiratory system-related diagnoses (Supplementary Table 2) and non-respiratory system-related diagnoses (Supplementary Table 3). Encounters in which patients had any of these diagnoses were not evaluated for appropriateness and were excluded from the inappropriate prescribing rate. Moreover, encounters in which patients had a non-respiratory system–related diagnosis were excluded from ARI prescribing rates.

### Measuring the inappropriate prescribing rate

For every included encounter, prescribed medications were extracted and classified by therapeutic and pharmaceutical class. Only systemic antibiotics were included (any topical, ophthalmic, and otic antibiotic preparations were excluded), and only prescriptions written for discharged patients were included (antibiotics given in the ED were excluded). For each attending physician the inappropriate prescribing rate was calculated as the number of included encounters with an antibiotic prescription divided by the total number of included encounters. An attending physician must have had at least 5 encounters to calculate a rate for any period. Any attending physicians with <5 encounters in the 6 months prior to e-mail distribution did not receive e-mails with their prescribing rates. Rates for attending physicians were calculated separately for episodes when they were working alone and with residents. In the former case, their rate only included encounters in which (1) they were the primary clinician of record (the discharge clinician who was also the clinician assigned for the longest time) and (2) no residents, fellows, physician assistants (PAs), or nurse practitioners (NPs) were documented in the patient’s chart. In the latter case, their rate included both encounters in which they were the primary clinician of record and those in which there was at least 1 resident (but no fellows, PAs, or NPs) in the chart whom they were supervising. Both rates were available on the dashboard, but the rate used for e-mail feedback was based on all encounters (including residents and when working alone). Encounters in which the record indicated treatment was provided by PAs or NPs were excluded from feedback because, unlike residents, PAs and NPs largely practice independently in ARI encounters in the ED. Encounters in which there was no clear primary clinician were not counted toward a specific attending physician’s rates, but along with encounters with PAs and NPs, were counted in the overall prescribing rate for the department.

In addition to the inappropriate prescribing rate, an overall ARI prescribing rate was calculated as the proportion of encounters with a diagnosis for a ARI (Supplementary Tables 1 and 2) that did not have a secondary non-ARI code (Supplementary Table 3) in which an antibiotic was prescribed at discharge. Similarly, the overall prescribing rates for bronchitis (in the absence of a chronic obstructive pulmonary disease indication), pharyngitis, and sinusitis (ie, diagnoses that have clear, evidence-based guidelines for when antibiotics are appropriate) were also calculated, and prescriptions for these diagnoses were included in the overall ARI prescribing rates.

### Peer comparison with behavioral feedback intervention

We created an automated algorithm that identified the prescribing attending physicians and biannually sent template-based e-mails (see example in Supplementary Fig. 1) to each individual attending physician with their inappropriate antibiotic prescribing rate. For attending physicians, we created a feedback dashboard (Supplementary Fig. 2) that was updated daily containing a chart depicting each attending physician’s rate. This dashboard allowed users to visualize and assess their own inappropriate prescribing rates in relation to those of their peers (users were blinded to the names of other prescribers). As in the study by Meeker et al,^7^ clinicians were specifically noted either to be in the top 10% of performers (clinicians with the lowest prescribing rates) or not to be among the 10% best performers. Because of improvements in prescribing over time, the group of 10% that performed best was expanded to the 25% of attending physicians that performed best during the latter half of the intervention because the proportion of attending physicians with very low rates (zero or 1 inappropriate prescription) was >10%. The dashboard also displayed a chart showing prescribing rates stratified by diagnosis category, in addition to patient encounter details that allowed attending physicians to look up specific cases in the EHR system. Built-in filters allowed users to compare their data with attending physicians in their own and different departments over variable periods of their choosing. Any attending physician with <5 encounters during the selected period did not appear on the dashboard for comparison. To further incentivize engagement by attending physicians, attending physicians’ review of their personal data was structured to satisfy the requirements for the American Board of Emergency Medicine Maintenance of Certification Improvement in Medical Practice Requirements.

### Statistical analysis

To assess the impact of the intervention on antibiotic prescribing between intervention and control sites, we used an interrupted time series design.^15–17^ A subset of clinicians worked at >1 of the intervention EDs; thus, the intervention sites were grouped for comparison to the control EDs. There was no clinician overlap between the intervention and control EDs. In addition to evaluating changes in antibiotic prescribing for ARI visits in which antibiotics are not appropriate, all respiratory encounters without a secondary exclusion code were assessed to ensure that any intervention impact was not just manifested as a change in coding. Although the intervention was targeted at attending physicians rather than PAs or NPs, we assessed the impact of the intervention on prescribing by attending physicians when no PA/NP was part of the care team, as well as in total in the department. The latter analysis was conducted because the PAs and NPs may have been aware of the ongoing feedback, and although they typically practice without significant oversight, the attending physicians are the physicians of record and may have influenced prescribing by the PAs and NPs. We fit the model using Prais-Winsten and robust standard errors to correct for first-order autocorrelation. In addition, because the number of ARI encounters that attending physicians see likely influences their prescribing patterns, we controlled for monthly ARI encounters and included a seasonal covariate to account for influenza season running from November to March. The lincom command was used to assess the difference between the postestimation trends for the intervention and control EDs. A difference-in-difference analysis was also run with the same covariates. All analyses were conducted using Stata version 14.2 software (StataCorp, College Station, TX).

## Results

During the study period, 51,928 ARI encounters did not have a secondary exclusion code (Supplementary Table 4). Of the ARI encounters, 28,544 were with attending physicians only and were included in the analysis. Overall, 38% of the attending physician–related ARI encounters occurred during the postintervention period, and 57% were at the intervention hospitals. The population at the intervention hospitals was younger, and they tended to live in more socioeconomically disadvantaged neighborhoods (Table 2). We detected no other differences in the demographics at each site between the pre- and postintervention periods. The cohorts included in the attending physician–only encounters and the entire ARI population (Supplementary Table 4) were similar.

**Table 2.** Demographics

Characteristic	Preintervention		Postintervention	
	Control	Intervention	Control	Intervention
ARI encounters (n = 28,544), no.	7,240	10,325	5,099	5,880
Age, mean, y	52.5	42.9	51.9	43.5
Sex, female, %	60.6	58.5	60.9	57.1
ARI prescribing rate, %	38.1	35.9	40.6	30.6
ARI encounters where antibiotics are inappropriate, %^a^	65.4	65.6	60.9	64.6
ARI encounters where antibiotics are or may be appropriate, %^a^	30.9	29.9	34.7	31.5
ADI, mean	3.3	6.9	3.5	6.8
No. of attending physicians	41	116	45	114

Note. ADI, area depravation index^19,20^; ARI, upper respiratory infection.

a These rows do not sum to 100% in a given column because encounters with a secondary exclusion code listed in Supplementary Table 3 were excluded.

### Overall ARI prescribing rates

**Fig. 1:**
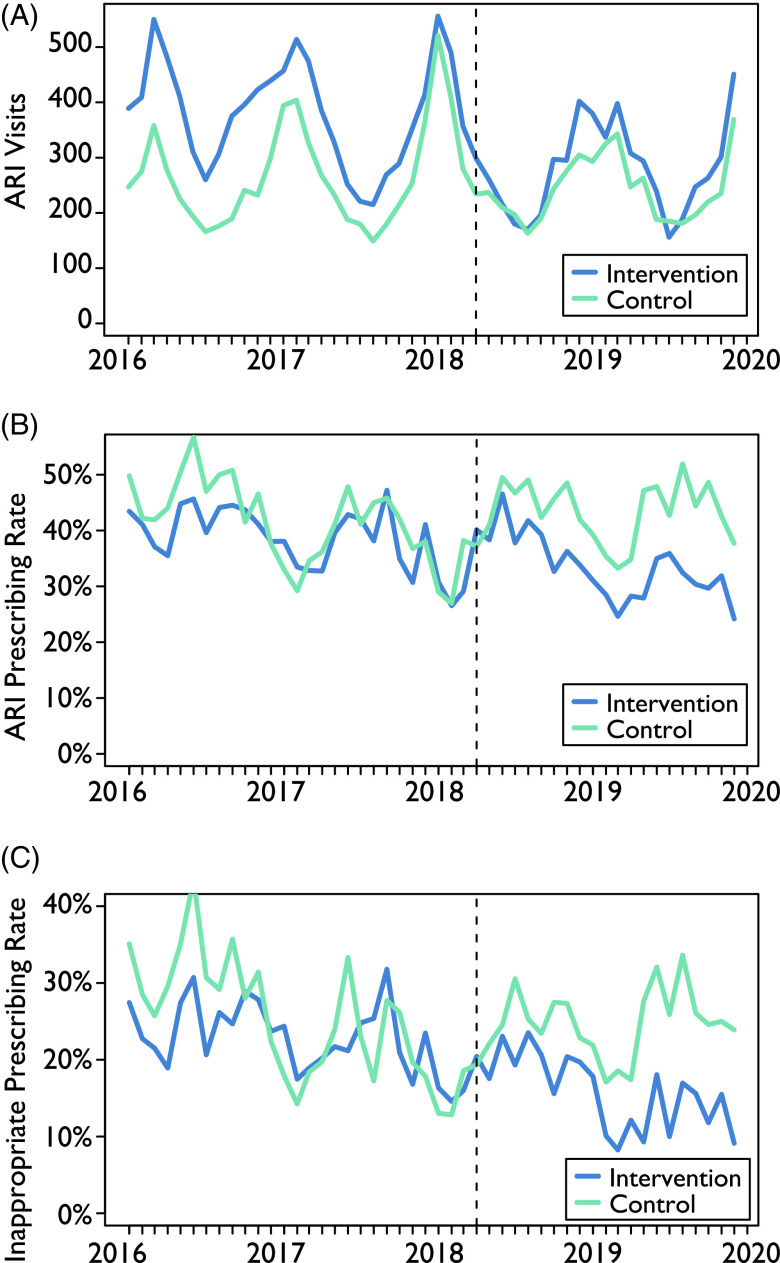
Upper respiratory infection visits and prescribing rates for intervention and control emergency departments. The intervention was implemented in the intervention emergency departments in April 2018 (vertical dashed line). The number of upper respiratory infection (ARI) visits (A) is seasonal, reflecting the increased likelihood of respiratory infections in the winter. The overall ARI prescribing rate (B) generally tends to follow an inverse seasonal pattern, reflecting an increased likelihood to prescribe when the number of ARI visits are lower, whereas the inappropriate prescribing rate (C) has only minor fluctuations and does not seem particularly related to season.

The overall ARI prescribing rates were 39.1% in the control hospitals and 34.0% in the intervention hospitals across the entire study timeline. Across the 2 periods, the prescribing rates were 38.1% and 40.6% at the control hospitals in the pre- and post-intervention periods, respectively. At the intervention hospitals, these rates were 35.9% and 30.6%, respectively (Table 2). However, these averages mask the seasonal differences and trends in prescribing observed over the course of the study (Fig. 1).

### Inappropriate ARI prescribing rates

**Fig. 2:**
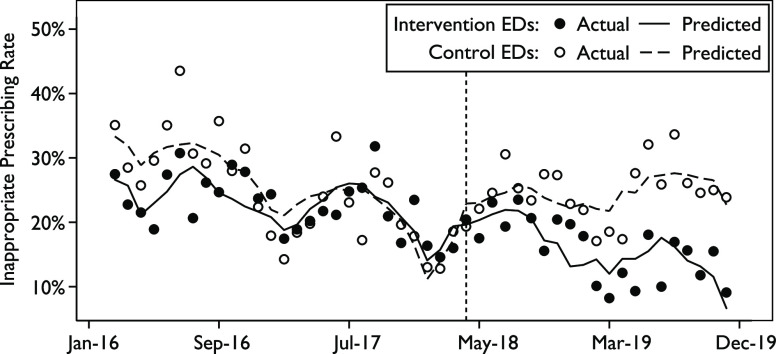
Interrupted time series regression analysis. The intervention was implemented in the intervention emergency departments in April 2018 (vertical dashed line).

Similar to the overall rates, the inappropriate prescribing rate remained stable at the control hospitals (23.0% and 23.8% in the pre- and post-intervention periods, respectively), and the rate decreased from 22.0% to 15.2% at the intervention hospitals (Table 3). In the intervention group, prescribing rates decreased across all diagnostic categories, and the largest decline occurred in bronchitis (11.2%). The control hospitals saw decreases in some categories and increases in others. For ARI encounters in which antibiotics are or may be appropriate, only the intervention hospitals had a decline, which was primarily due to a decline in prescribing for pharyngitis. During the pre-intervention period, the inappropriate prescribing rate at the control hospitals, which was ∼10% higher initially, fell during the first half of the period and then stabilized at a rate similar to that of the intervention hospitals. However, after the intervention began, the rates between the sites diverged to a difference of ∼10% (Fig. 2).

**Table 3.** Antibiotic Prescribing by Encounter Type and Diagnosis

Characteristic	Preintervention		Postintervention	
	Control	Intervention	Control	Intervention
**Encounters in which antibiotics are inappropriate, no. (% prescribed)**	4,737 (23.0)	6,777 (22.0)	3,105 (23.8)	3,800 (15.2)
AURI^a^	717 (17.0)	1,490 (22.7)	446 (26.5)	826 (13.7)
Asthma	593 (18.2)	1,312 (11.4)	422 (14.0)	752 (7.0)
Bronchitis	1,039 (55.5)	1,198 (63.9)	694 (52.7)	535 (49.2)
Cough	727 (27.1)	741 (19.3)	676 (20.0)	573 (16.9)
Other^b^	1,661 (5.1)	2,036 (4.8)	867 (7.2)	1,114 (4.6)
**ARI encounters in which antibiotics are or may be appropriate, no. (% prescribed)**	2,237 (70.0)	3,085 (66.4)	1,770 (70.1)	1,854 (62.2)
Bacterial/chronic bronchitis and COPD	263 (41.4)	459 (49.0)	183 (47.5)	281 (49.8)
Pharyngitis	560 (46.6)	1098 (48.8)	488 (39.5)	717 (40.3)
Pneumonia	904 (89.3)	775 (93.7)	755 (92.7)	458 (94.1)
Sinusitis	391 (76.0)	562 (72.6)	255 (74.1)	291 (71.5)
Other^c^	119 (78.2)	191 (80.6)	89 (79.8)	107 (80.4)

Note. ARI, acute respiratory infection; AURI, acute upper respiratory infection; COPD, chronic obstructive pulmonary disease.

a See Supplementary Table 1.

b Includes influenza, viral pneumonia, and other diagnoses shown in Supplementary Table 1.

c Includes influenza with pneumonia, tonsillitis, and other ARI diagnoses shown in Supplementary Table 2.

Consistent with these observations, there was no significant difference in the prescribing rate or trend between the control and intervention EDs during the pre-intervention period (Table 4). In the post-intervention period, the difference in monthly trends in the effect of the intervention between groups was negative and significant (−0.009; 95% CI, −0.013 to −0.004; *P* < .001), reflecting a greater decrease in the prescribing rate in the intervention group (Table 4). The estimated post-intervention linear trends between groups were also significantly different (−0.006; 95% CI, −0.010 to −0.003; *P* < .001) (Table 4). The difference-in-difference analysis similarly detected a significant decrease in the prescribing rate after introduction of the intervention (−0.06; 95% CI, −0.11 to −0.02; *P* < .01).

**Table 4.** Interrupted Time Series Regression Analysis of Inappropriate Prescribing for Upper Respiratory Infections after Introduction of Peer Comparison and Feedback in Intervention and Control Emergency Departments

Variable	Result (95% CI)	*P* Value
ARI visits	−0.00030 (−0.00043 to −0.00016)	<.001
Flu season (November–March)	−0.00066 (−0.02202 to 0.02070)	.95
Preintervention trend	−0.006 (−0.008 to −0.004)	<.001
Initial difference between intervention and control groups	−0.025 (−0.077 to 0.027)	.34
Preintervention trend difference	0.003 (0.000–0.006)	.06
Immediate intervention impact	0.049 (0.004–0.093)	.03
Postintervention trend	0.008 (0.005–0.011)	<.001
Immediate intervention impact difference	−0.061 (−0.118 to −0.004)	.04
Postintervention trend difference	−0.009 (−0.013 to −0.004)	<.001
Constant	0.408 (0.363–0.452)	<.001
**Linear change**		
Intervention group	−0.004 (−0.007 to −0.002)	<.001
Control group	0.002 (−0.001 to 0.004)	.12
Difference	−0.006 (−0.010 to −0.003)	<.001

Note. ARI, acute respiratory infection; CI, confidence interval.

### Infection type-specific ARI prescribing rates

Because the intervention was targeted at the results of encounters measured by ICD-10 codes, the change in inappropriate prescribing could have been due to attending physicians shifting from antibiotic-inappropriate diagnosis codes (eg, bronchitis) to antibiotic-appropriate diagnosis codes (eg, pharyngitis). To account for this possibility, we conducted a similar interrupted time series analysis for bronchitis, pharyngitis, and sinusitis at the control hospitals to assess whether any of them had increased after introduction of the intervention. No significant trend was observed in any of these diagnoses.

## Discussion

In this multicenter, quasi-experimental, quality improvement project we implemented a behavioral feedback intervention utilizing clinician feedback with peer comparison to target antibiotic prescribing for ARIs in attending physicians in the ED. The inappropriate prescribing rate remained stable in the control group but decreased significantly at the intervention sites between the pre- and post-intervention periods. These findings suggest that implementation of the intervention was associated with improved antibiotic prescribing for ARIs.

Although behavioral feedback interventions targeting antibiotic use for ARIs have been associated with reduced antibiotic prescribing for adults in the outpatient setting,^7,12^ their potential in the ED has been understudied. One study demonstrated the potential for an intervention incorporating behavioral feedback to decrease antibiotic prescribing for ARIs in the ED.^18^ As in our intervention, clinicians were sent e-mails with their inappropriate ARI prescribing rate so they could compare their performance to that of their top-performing peers. However, the behavioral feedback was part of a bundled educational package that was implemented at all sites, which limited the ability to compare effectiveness. Similarly, in another study, a behavioral feedback intervention was conducted at 2 EDs within the Veterans’ Health Administration.^14^ In this case, targeted clinicians received initial one-on-one feedback on their antimicrobial prescribing patterns as well as education on prescribing guidelines. Continued quarterly feedback (primarily via e-mail) on uncomplicated ARI and total antimicrobial use was then continued for the duration of the study. The implementation of the initiative was associated with reduced inappropriate antimicrobial use for uncomplicated ARIs. However, the intervention was targeted at clinicians who were considered amenable and involved extensive initial face-to-face feedback.

Although both prior interventions suggest that behavioral feedback with peer comparison may work, they were largely combined with other intensive actions such that the ability to judge implementation of electronic feedback alone is limited. Our results are based solely on the implementation of an automated behavioral feedback intervention with the only incentive to participate being maintenance of certification credit, allowing a more direct attribution of the observed reduction in inappropriate prescribing to the behavioral feedback approach in the adult ED population. In addition, the rate of inappropriate prescribing at the 3 intervention sites was compared to the rate at 2 control sites, strengthening the conclusion that implementation of behavioral feedback may successfully reduce inappropriate prescribing for ARIs in the ED.

Although the trends are robust and our study has strengths, there remain several limitations. First, by using ICD-10 codes to identify encounters for inclusion, it is possible that we missed relevant encounters due to a missing diagnosis code for a respiratory condition that warrants or may warrant antibiotics. However, we have no indication that ICD-10 codes were more likely to be missing from the intervention versus the control group. In addition, instead of changing prescribing patterns, clinicians could have changed coding practices. However, it is unlikely that the noted decrease can be explained by attending physicians shifting their use of diagnosis codes because we detected no significant trends in the numbers of bronchitis, pharyngitis, and sinusitis diagnoses. Second, though the target and control EDs are located in the same geographic area, they draw from different patient populations, which may bias decision making. Third, though we do not know of anything specific, in the context of national attention to the topic of inappropriate prescribing and antibiotic stewardship, other initiatives to change prescribing may have also been implemented at the hospitals differentially. Fourth, while all EDs were part of the same health system, laboratory methods for identifying infections, such as rapid influenza tests, may have differed over time and among EDs. In addition, hospital-specific factors, such as hospital type (ie, academic vs community), number of residents, presence of PAs and/or NPs, and potentially different antimicrobial stewardship commitments, could have biased the results. Fifth, the intervention focused only on attending physicians and not other clinicians, and we examined only antibiotic prescribing without adjudicating antimicrobial selection or duration of prescribing for conditions requiring antibiotics. Finally, while the presumed mechanism driving changes in prescribing was peer comparison, the fact that attending physicians knew they were being observed on their prescribing habits (Hawthorne effect) may also have contributed to the outcome.

Our results suggest that behavioral feedback can be successful in the adult ED at reducing antibiotic use. Although other studies have shown that peer comparison can be effective, they have also shown that without continued intervention the impact on prescribing is not sustained.^7–9^ Using an automated system implemented as a quality improvement project rather than a research study, our initiative can continue with only limited resources, which we hope will allow the intervention to remain effective. As behavioral feedback that provides simple comparisons to others becomes a more accepted method for modifying clinicians’ practices, further applications of this approach can be explored to reduce inappropriate prescribing, combat the spread of resistance, and improve patient outcomes.

## Supplementary material

For supplementary material accompanying this paper visit https://doi.org/10.1017/ash.2021.240. click here to view supplementary material
