# Supplementary material for: Improving antimicrobial prescribing for upper respiratory infections in the emergency department: Implementation of peer comparison with behavioral feedback
Source: Antimicrob Steward Healthc Epidemiol. 2021 Dec 23;1(1):e70. doi: 10.1017/ash.2021.240 (PMC9495637; doi:10.1017/ash.2021.240)
Supplement: Supplementary file 1 [file S2732494X21002400sup001.docx]

**Supplementary Material**

**for**

**Improving antimicrobial prescribing for upper respiratory infections in the emergency department: implementation of peer comparison with behavioral feedback**

George F. Jones, BS^1,2^, Valeria Fabre, MD^1^, Jeremiah Hinson, MD, PhD^3^, Scott Levin, PhD^3^, Matthew Toerper, MSc^3^, Jennifer Townsend, MD^4^, Sara E. Cosgrove, MD^1^, Mustapha Saheed, MD^3^, Eili Y. Klein, PhD^3,5^

^1^Department of Medicine, Division of Infectious Diseases, Johns Hopkins University School of Medicine, Baltimore, Maryland, USA

^2^Eastern Virginia Medical School, Norfolk, Virginia, USA

^3^Department of Emergency Medicine, Johns Hopkins University School of Medicine, Baltimore, Maryland, USA

^4^Division of Infectious Diseases, Greater Baltimore Medical Center, Towson, Maryland, USA

^5^Center for Disease Dynamics, Economics & Policy, Washington DC, USA


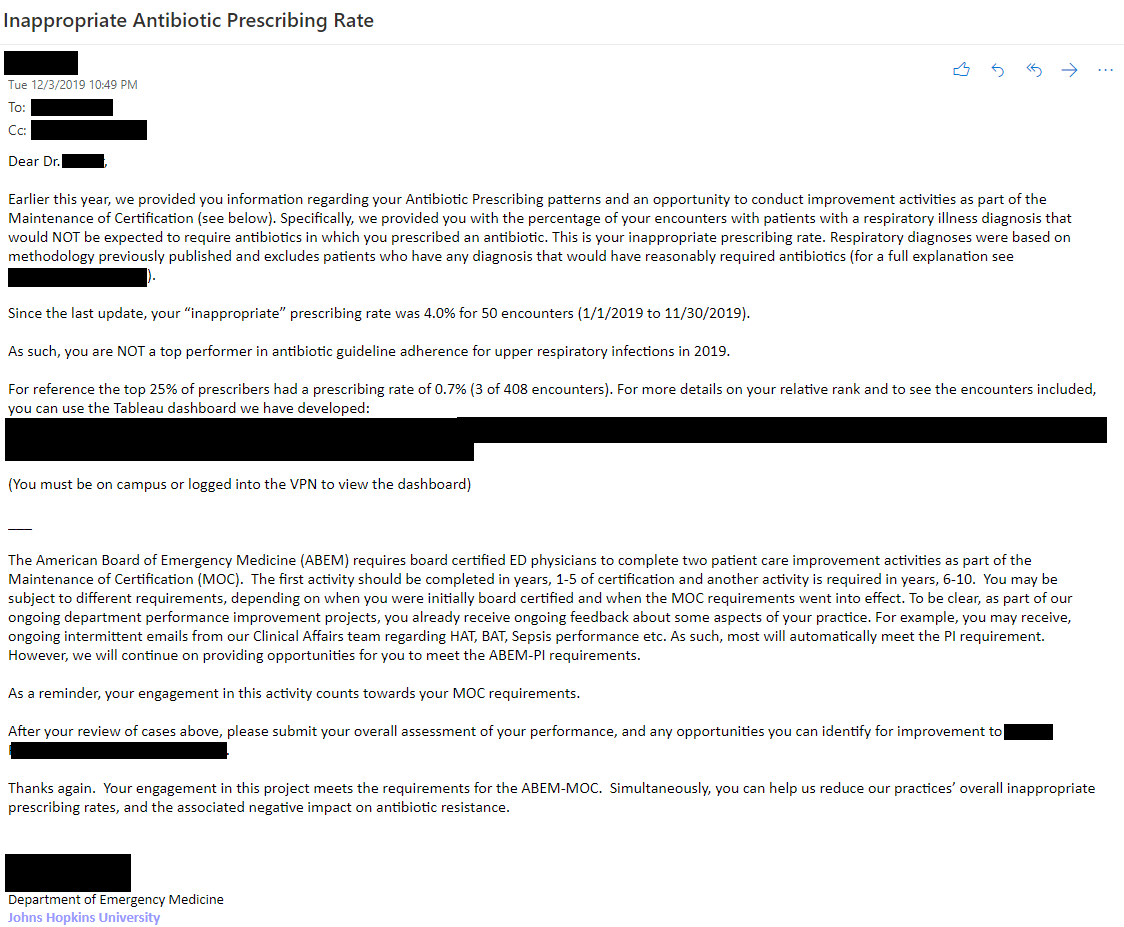


**Supplementary Figure 1. Example feedback email.**

Example email sent to providers on an approximately biannual basis providing them with feedback on their inappropriate prescribing rate. Provider-specific information has been redacted to maintain confidentiality.

**
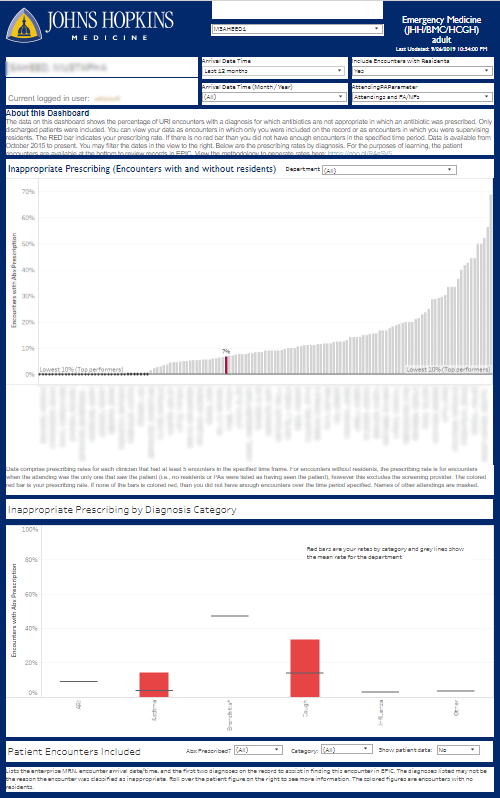
**

**Supplementary Figure 2. Provider feedback dashboard.**

Providers in the top 10% are shown for peer comparison. Rolling over each column shows the percentage for each provider and the number of encounters on which the rate of inappropriate prescribing is based. Filters allow the provider to compare data over different timelines and across departments.

| **Supplementary Table 1. Diagnosis codes used to categorize respiratory condition encounters where antibiotics are inappropriate.** | | |
| --- | --- | --- |
| **ICD-10 Diagnosis Code** | **Diagnosis** | **Category** |
| J00 | Acute nasopharyngitis [common cold] | AURI |
| J04.0 | Acute laryngitis | AURI |
| J04.2 | Acute laryngotracheitis | AURI |
| J06.0 | Acute laryngopharyngitis | AURI |
| J06.9 | Acute upper respiratory infection, unspecified | AURI |
| J09.X2 | Influenza due to identified novel influenza A virus | Influenza |
| J10.1 | Influenza due to other identified influenza virus | Influenza |
| J10.2 | Influenza due to other identified influenza virus | Influenza |
| J11.1 | Influenza due to unidentified influenza virus with other resp manifest | Influenza |
| J11.2 | Influenza due to unidentified influenza virus with GI manifest | Influenza |
| J11.89 | Influenza due to unidentified influenza virus with other manifest | Influenza |
| J20.3 | Acute bronchitis due to coxsackievirus | Bronchitis^a^ |
| J20.4 | Acute bronchitis due to parainfluenza virus | Bronchitis^a^ |
| J20.5 | Acute bronchitis due to respiratory syncytial virus | Bronchitis^a^ |
| J20.6 | Acute bronchitis due to rhinovirus | Bronchitis^a^ |
| J20.7 | Acute bronchitis due to echovirus | Bronchitis^a^ |
| J20.8 | Acute bronchitis due to other specified organisms | Bronchitis^a^ |
| J20.9 | Acute bronchitis, unspecified | Bronchitis^a^ |
| J21.0 | Acute bronchiolitis due to respiratory syncytial virus | Bronchitis^a^ |
| J21.1 | Acute bronchiolitis due to human metapneumovirus | Bronchitis^a^ |
| J21.8 | Acute bronchiolitis due to other specified organisms | Bronchitis^a^ |
| J21.9 | Acute bronchiolitis, unspecified | Bronchitis^a^ |
| J40 | Bronchitis, not specified as acute or chronic | Bronchitis^a^ |
| J30.0 | Vasomotor rhinitis | Other |
| J30.1 | Allergic rhinitis due to pollen | Other |
| J30.2 | Other seasonal allergic rhinitis | Other |
| J30.5 | Allergic rhinitis due to food | Other |
| J30.81 | Allergic rhinitis due to animal (cat) (dog) hair a | Other |
| J30.89 | Other allergic rhinitis | Other |
| J30.9 | Allergic rhinitis, unspecified | Other |
| J31.0 | Chronic rhinitis | Other |
| J31.1 | Chronic nasopharyngitis | Other |
| J33.0 | Polyp of nasal cavity | Other |
| J33.1 | Polypoid sinus degeneration | Other |
| J33.8 | Other polyp of sinus | Other |
| J33.9 | Nasal polyp, unspecified | Other |
| J34.1 | Cyst and mucocele of nose and nasal sinus | Other |
| J34.2 | Deviated nasal septum | Other |
| J34.3 | Hypertrophy of nasal turbinates | Other |
| J34.81 | Nasal mucositis (ulcerative) | Other |
| J34.89 | Other specified disorders of nose and nasal sinuse | Other |
| J34.9 | Unspecified disorder of nose and nasal sinuses | Other |
| J35.01 | Chronic tonsillitis | Other |
| J35.02 | Chronic adenoiditis | Other |
| J35.03 | Chronic tonsillitis and adenoiditis | Other |
| J35.1 | Hypertrophy of tonsils | Other |
| J35.2 | Hypertrophy of adenoids | Other |
| J35.3 | Hypertrophy of tonsils with hypertrophy of adenoid | Other |
| J35.8 | Other chronic diseases of tonsils and adenoids | Other |
| J35.9 | Chronic disease of tonsils and adenoids, unspecifi | Other |
| J37.0 | Chronic laryngitis | Other |
| J37.1 | Chronic laryngotracheitis | Other |
| J38.00 | Paralysis of vocal cords and larynx, unspecified | Other |
| J38.01 | Paralysis of vocal cords and larynx, unilateral | Other |
| J38.02 | Paralysis of vocal cords and larynx, bilateral | Other |
| J38.1 | Polyp of vocal cord and larynx | Other |
| J38.2 | Nodules of vocal cords | Other |
| J38.3 | Other diseases of vocal cords | Other |
| J38.4 | Edema of larynx | Other |
| J38.5 | Laryngeal spasm | Other |
| J38.6 | Stenosis of larynx | Other |
| J38.7 | Other diseases of larynx | Other |
| J39.2 | Other diseases of pharynx | Other |
| J39.3 | Upper respiratory tract hypersensitivity reaction, site unsp | Other |
| J39.8 | Other specified diseases of upper respiratory tract | Other |
| J39.9 | Disease of upper respiratory tract, unspecified | Other |
| B34.9 | Viral infection, unspecified | Other |
| R09.81 | Nasal congestion | Other |
| R09.82 | Postnasal drip | Other |
| B97.4 | Respiratory syncytial virus as the cause of disease classified elsewhere | Other |
| J12.9 | Viral pneumonia, unspecified | Pneumonia |
| J45.20 | Mild intermittent asthma, uncomplicated | Asthma |
| J45.21 | Mild intermittent asthma with (acute) exacerbation | Asthma |
| J45.30 | Mild persistent asthma, uncomplicated | Asthma |
| J45.31 | Mild persistent asthma with (acute) exacerbation | Asthma |
| J45.40 | Moderate persistent asthma, uncomplicated | Asthma |
| J45.41 | Moderate persistent asthma with (acute) exacerbation | Asthma |
| J45.901 | Unspecified asthma with (acute) exacerbation | Asthma |
| J45.909 | Unspecified asthma, uncomplicated | Asthma |
| J45.998 | Other asthma | Asthma |
| R05 | Cough | Cough |
| ^a^ No COPD indication either in current visit or prior visit (ICD-10 code: J44). | | |

| **Supplementary Table 2. Diagnosis codes for respiratory infections for which antibiotics are or may be appropriate.** | | |
| --- | --- | --- |
| **ICD-10 Diagnosis Code** | **Diagnosis** | **Category** |
| J20.0 | Acute bronchitis due to Mycoplasma pneumoniae | Bacterial Bronchitis |
| J20.1 | Acute bronchitis due to Hemophilus influenzae | Bacterial Bronchitis |
| J20.2 | Acute bronchitis due to streptococcus | Bacterial Bronchitis |
| J22 | Unspecified acute lower respiratory infection | Bacterial Bronchitis |
| J41.0 | Simple chronic bronchitis | Chronic Bronchitis |
| J41.1 | Mucopurulent chronic bronchitis | Chronic Bronchitis |
| J41.8 | Mixed simple and mucopurulent chronic bronchitis | Chronic Bronchitis |
| J42 | Unspecified chronic bronchitis | Chronic Bronchitis |
| J44.0 | Chronic obstructive pulmon disease w acute lower resp infct | COPD |
| J44.1 | Chronic obstructive pulmonary disease w (acute) exacerbation | COPD |
| J44.9 | Chronic obstructive pulmonary disease, unspecified | COPD |
| J09.X1 | Influenza due to ident novel influenza A virus w pneumonia | Influenza with Pneumonia |
| J09.X9 | Flu due to ident novel influenza A virus w oth manifest | Influenza with Pneumonia |
| J10.00 | Flu due to oth ident flu virus w unsp type of pneumonia | Influenza with Pneumonia |
| J10.01 | Flu due to oth ident flu virus w same oth ident flu virus pn | Influenza with Pneumonia |
| J10.08 | Influenza due to oth ident influenza virus w oth pneumonia | Influenza with Pneumonia |
| J10.81 | Influenza due to oth ident influenza virus w encephalopathy | Influenza with Pneumonia |
| J10.82 | Influenza due to oth ident influenza virus w myocarditis | Influenza with Pneumonia |
| J10.83 | Influenza due to oth ident influenza virus w otitis media | Influenza with Pneumonia |
| J10.89 | Influenza due to oth ident influenza virus w oth manifest | Influenza with Pneumonia |
| J11.00 | Flu due to unidentified flu virus w unsp type of pneumonia | Influenza with Pneumonia |
| J11.08 | Flu due to unidentified flu virus w specified pneumonia | Influenza with Pneumonia |
| J11.81 | Flu due to unidentified influenza virus w encephalopathy | Influenza with Pneumonia |
| J11.82 | Influenza due to unidentified influenza virus w myocarditis | Influenza with Pneumonia |
| J11.83 | Influenza due to unidentified influenza virus w otitis media | Influenza with Pneumonia |
| J04.10 | Acute tracheitis without obstruction | Other ARI |
| J04.11 | Acute tracheitis with obstruction | Other ARI |
| J04.30 | Supraglottitis, unspecified, without obstruction | Other ARI |
| J34.0 | Abscess, furuncle and carbuncle of nose | Other ARI |
| J39.0 | Retropharyngeal and parapharyngeal abscess | Other ARI |
| J39.1 | Other abscess of pharynx | Other ARI |
| J02.0 | Streptococcal pharyngitis | Pharyngitis |
| J02.8 | Acute pharyngitis due to other specified organisms | Pharyngitis |
| J02.9 | Acute pharyngitis, unspecified | Pharyngitis |
| J03.00 | Acute streptococcal tonsillitis, unspecified | Pharyngitis |
| J03.01 | Acute recurrent streptococcal tonsillitis | Pharyngitis |
| J31.2 | Chronic pharyngitis | Pharyngitis |
| J13 | Pneumonia due to Streptococcus pneumoniae | Pneumonia |
| J14 | Pneumonia due to Hemophilus influenzae | Pneumonia |
| J15.0 | Pneumonia due to Klebsiella pneumoniae | Pneumonia |
| J15.1 | Pneumonia due to Pseudomonas | Pneumonia |
| J15.20 | Pneumonia due to staphylococcus, unspecified | Pneumonia |
| J15.21 | Pneumonia due to staphylococcus aureus | Pneumonia |
| J15.211 | Pneumonia due to methicillin suscep staph | Pneumonia |
| J15.212 | Pneumonia due to Methicillin resistant Staphylococcus aureus | Pneumonia |
| J15.29 | Pneumonia due to other staphylococcus | Pneumonia |
| J15.3 | Pneumonia due to streptococcus, group B | Pneumonia |
| J15.4 | Pneumonia due to other streptococci | Pneumonia |
| J15.5 | Pneumonia due to Escherichia coli | Pneumonia |
| J15.6 | Pneumonia due to other aerobic Gram-negative bacteria | Pneumonia |
| J15.7 | Pneumonia due to Mycoplasma pneumoniae | Pneumonia |
| J15.8 | Pneumonia due to other specified bacteria | Pneumonia |
| J15.9 | Unspecified bacterial pneumonia | Pneumonia |
| J16.0 | Chlamydial pneumonia | Pneumonia |
| J16.8 | Pneumonia due to other specified infectious organisms | Pneumonia |
| J17 | Pneumonia in diseases classified elsewhere | Pneumonia |
| J18.0 | Bronchopneumonia, unspecified organism | Pneumonia |
| J18.1 | Lobar pneumonia, unspecified organism | Pneumonia |
| J18.2 | Hypostatic pneumonia, unspecified organism | Pneumonia |
| J18.8 | Other pneumonia, unspecified organism | Pneumonia |
| J18.9 | Pneumonia, unspecified organism | Pneumonia |
| J01.00 | Acute maxillary sinusitis, unspecified | Sinusitis |
| J01.01 | Acute recurrent maxillary sinusitis | Sinusitis |
| J01.10 | Acute frontal sinusitis, unspecified | Sinusitis |
| J01.11 | Acute recurrent frontal sinusitis | Sinusitis |
| J01.20 | Acute ethmoidal sinusitis, unspecified | Sinusitis |
| J01.21 | Acute recurrent ethmoidal sinusitis | Sinusitis |
| J01.30 | Acute sphenoidal sinusitis, unspecified | Sinusitis |
| J01.31 | Acute recurrent sphenoidal sinusitis | Sinusitis |
| J01.40 | Acute pansinusitis, unspecified | Sinusitis |
| J01.41 | Acute recurrent pansinusitis | Sinusitis |
| J01.80 | Other acute sinusitis | Sinusitis |
| J01.81 | Other acute recurrent sinusitis | Sinusitis |
| J01.90 | Acute sinusitis, unspecified | Sinusitis |
| J01.91 | Acute recurrent sinusitis, unspecified | Sinusitis |
| J32.0 | Chronic maxillary sinusitis | Sinusitis |
| J32.1 | Chronic frontal sinusitis | Sinusitis |
| J32.2 | Chronic ethmoidal sinusitis | Sinusitis |
| J32.3 | Chronic sphenoidal sinusitis | Sinusitis |
| J32.4 | Chronic pansinusitis | Sinusitis |
| J32.8 | Other chronic sinusitis | Sinusitis |
| J32.9 | Chronic sinusitis, unspecified | Sinusitis |
| J03.80 | Acute tonsillitis due to other specified organisms | Tonsillitis |
| J03.81 | Acute recurrent tonsillitis due to other specified organisms | Tonsillitis |
| J03.90 | Acute tonsillitis, unspecified | Tonsillitis |
| J03.91 | Acute recurrent tonsillitis, unspecified | Tonsillitis |
| J36 | Peritonsillar abscess | Tonsillitis |

| **Supplementary Table 3. Diagnosis codes for non-respiratory infections for which antibiotics are/may be appropriate.** | |
| --- | --- |
| **ICD-10 Diagnosis Code** | **Diagnosis** |
| A04 | Other bacterial intestinal infections |
| A05 | Other bacterial foodborne intoxications, not elsewhere classified |
| A06.2 | Amoebic nondysenteric colitis |
| A09 | Infectious gastroenteritis and colitis, unspecified |
| A15 | Respiratory tuberculosis |
| A17 | Tuberculosis of nervous system |
| A18 | Tuberculosis of other organs |
| A19 | Miliary tuberculosis |
| A20 | Plague |
| A21 | Tularemia |
| A22 | Anthrax |
| A23 | Brucellosis |
| A24 | Glanders and melioidosis |
| A25 | Rat-bite fevers |
| A26 | Erysipeloid |
| A27 | Leptospirosis |
| A28 | Other zoonotic bacterial diseases, not elsewhere classified |
| A30 | Leprosy [Hansen's disease] |
| A31 | Infection due to other mycobacteria |
| A32 | Listeriosis |
| A33 | Tetanus neonatorum |
| A34 | Obstetrical tetanus |
| A35 | Other tetanus |
| A36 | Diphtheria |
| A37 | Whooping cough |
| A38 | Scarlet fever |
| A39 | Meningococcal meningitis |
| A40 | Streptococcal sepsis |
| A41 | Other sepsis |
| A42 | Actinomycosis |
| A43 | Nocardiosis |
| A44 | Bartonellosis |
| A46 | Erysipelas |
| A48 | Other bacterial diseases, not elsewhere classified |
| A49 | Bacterial infection of unspecified site |
| A50 | Congenital syphilis |
| A51 | Early syphilis |
| A52 | Late syphilis |
| A53 | Other and unspecified syphilis |
| A54 | Gonococcal infection |
| A55 | Chlamydial lymphogranuloma (venereum) |
| A56 | Other sexually transmitted chlamydial diseases |
| A57 | Chancroid |
| A58 | Granuloma inguinale |
| A59 | Trichomoniasis |
| A64 | Unspecified sexually transmitted disease |
| A65 | Nonvenereal syphilis |
| A66 | Yaws |
| A67 | Pinta [carate] |
| A68 | Relapsing fevers |
| A69 | Other spirochetal infections |
| A70 | Chlamydia psittaci infections |
| A71 | Trachoma |
| A74 | Other diseases caused by chlamydiae |
| A75 | Typhus fever |
| A77 | Spotted fever [tick-borne rickettsioses] |
| A78 | Q fever |
| A79 | Other rickettsioses |
| B58.2 | Toxoplasma meningoencephalitis |
| B95 | Streptococcus, Staphylococcus, and Enterococcus as the cause of diseases classified elsewhere |
| B96 | Other bacterial agents as the cause of diseases classified elsewhere |
| G00 | Bacterial meningitis, not elsewhere classified |
| G01 | Meningitis in bacterial diseases classified elsewh |
| G02 | Meningitis in other infectious and parasitic disea |
| G03 | Meningitis due to other and unspecified causes |
| G04 | Encephalitis, myelitis and encephalomyelitis |
| G05 | Encephalitis, myelitis and encephalomyelitis in diseases classified elsewhere |
| G06 | Intracranial and intraspinal abscess and granuloma |
| H00.03 | Abscess of eyelid |
| H05.01 | Cellulitis of orbit |
| H05.02 | Osteomyelitis of orbit |
| H05.03 | Periostitis of orbit |
| H60 | Otitis externa |
| H65 | Nonsuppurative otitis media |
| H66 | Suppurative and unspecified otitis media |
| I00 | Rheumatic fever without heart involvement |
| I01 | Rheumatic fever with heart involvement |
| I02 | Rheumatic chorea |
| I33 | Acute and subacute endocarditis |
| I96 | Gangrene, not elsewhere classified |
| J47 | Bronchiectasis |
| J95.851 | Ventilator associated pneumonia |
| K02 | Dental caries |
| K04.4 | Acute apical periodontitis of pulpal origin |
| K04.6 | Chronic apical periodontitis |
| K04.6 | Periapical abscess with sinus |
| K04.7 | Periapical abscess without sinus |
| K05.2 | Aggressive periodontitis |
| K05.3 | Chronic periodontitis |
| K05.4 | Periodontosis |
| K11.2 | Sialoadenitis |
| K12.2 | Cellulitis and abscess of mouth |
| K35 | Acute appendicitis |
| K50 | Crohn's disease |
| K52.1 | Toxic gastroenteritis and colitis |
| K57.2 | Diverticulitis of large intestine with perforation |
| K57.3 | Diverticulitis of large intestine without perforat |
| K61 | Abscess of anal and rectal regions |
| K65.0 | Generalized (acute) peritonitis |
| K65.1 | Peritoneal abscess |
| K65.2 | Spontaneous bacterial peritonitis |
| K65.9 | Peritonitis, unspecified |
| K68.1 | Retroperitoneal abscess |
| K75.0 | Abscess of liver |
| K80.0 | Calculus of gallbladder with acute cholecystitis |
| K80.18 | Calculus of gallbladder with other cholecystitis w |
| K80.19 | Calculus of gallbladder with other cholecystitis w |
| K80.42 | Calculus of bile duct with acute cholecystitis wit |
| K80.44 | Calculus of bile duct with chronic cholecystitis w |
| K81.0 | Acute cholecystitis |
| K83.0 | Cholangitis |
| K91.850 | Pouchitis |
| L00 | Staphylococcal scalded skin syndrome |
| L01.00 | Impetigo, unspecified |
| L01.09 | Other impetigo |
| L02 | Cutaneous abscess, furuncle and carbuncle |
| L03 | Cellulitis and acute lymphangitis |
| L04 | Acute lymphadenitis |
| L05.01 | Pilonidal cyst with abscess |
| L05.91 | Pilonidal cyst without abscess |
| L08.89 | Other specified local infections of the skin and s |
| L08.9 | Local infection of the skin and subcutaneous tissu |
| M00 | Pyogenic arthritis |
| M01 | Direct infections of joint in infectious and parasitic diseases classified elsewhere |
| M46.20 | Osteomyelitis of vertebra, site unspecified |
| M46.30 | Infection of intervertebral disc (pyogenic), site |
| M60.0 | Infective myositis |
| M86 | Osteomyelitis |
| N10 | Acute pyelonephritis |
| N12 | Tubulo-interstitial nephritis, not specified as ac |
| N28.84 | Pyelitis cystica |
| N30.0 | Acute cystitis |
| N30.20 | Other chronic cystitis without hematuria |
| N30.80 | Other cystitis without hematuria |
| N30.90 | Cystitis, unspecified without hematuria |
| N34.1 | Nonspecific urethritis |
| N34.2 | Other urethritis |
| N35.111 | Postinfective urethral stricture, not elsewhere cl |
| N39.0 | Urinary tract infection, site not specified |
| N41.0 | Acute prostatitis |
| N41.1 | Chronic prostatitis |
| N41.9 | Inflammatory disease of prostate, unspecified |
| N43.1 | Infected hydrocele |
| N45.1 | Epididymitis |
| N51 | Disorders of male genital organs in diseases class |
| N61 | Inflammatory disorders of breast |
| N72 | Inflammatory disease of cervix uteri |
| N73.0 | Acute parametritis and pelvic cellulitis |
| N73.3 | Female acute pelvic peritonitis |
| N73.9 | Female pelvic inflammatory disease, unspecified |
| N75.0 | Cyst of Bartholin's gland |
| N75.1 | Abscess of Bartholin's gland |
| N76.0 | Acute vaginitis |
| N76.4 | Abscess of vulva |
| N77.1 | Vaginitis, vulvitis and vulvovaginitis in diseases |
| O23 | Infections of genitourinary tract in pregnancy |
| O41.1010 | Infection of amniotic sac and membranes, unspecifi |
| O41.1090 | Infection of amniotic sac and membranes, unspecifi |
| O85 | Puerperal sepsis |
| O86 | Other puerperal infections |
| O91 | Infections of breast associated with pregnancy, the puerperium and lactation |
| P36 | Bacterial sepsis of newborn |
| P39.0 | Neonatal infective mastitis |
| P39.2 | Intra-amniotic infection affecting newborn, not el |
| P39.3 | Neonatal urinary tract infection |
| P39.4 | Neonatal skin infection |
| P39.8 | Other specified infections specific to the perinatal period |
| P39.9 | Infection specific to the perinatal period, unspecified |
| P77 | Necrotizing enterocolitis of newborn |
| R09.1 | Pleurisy |
| R50.81 | Fever presenting with conditions classified elsewh |
| R50.9 | Fever, unspecified |
| R78.81 | Bacteremia |
| R82.71 | Bacteriuria |
| S01 | Open wound of head |
| S11 | Open wound of neck |
| S21 | Open wound of thorax |
| S31 | Open wound of abdomen, lower back, pelvis and external genitals |
| S41 | Open wound of shoulder and upper arm |
| S51 | Open wound of elbow and forearm |
| S61 | Open wound of wrist, hand and fingers |
| S71 | Open wound of hip and thigh |
| S81 | Open wound of knee and lower leg |
| S91 | Open wound of ankle, foot and toes |
| T80.2 | Infections following infusion, transfusion and therapeutic injectio |
| W54.0XXA | Bitten by dog, initial encounter |
| Z20.1 | Contact with and (suspected) exposure to tuberculosis |
| Z20.2 | Contact with and (suspected) exposure to infections with a predominantly sexual mode of transmission |
| Z20.81 | Contact with and (suspected) exposure to other bacterial communicable diseases |
| Z20.89 | Contact with and (suspected) exposure to other communicable diseases |
| Z20.9 | Contact with and (suspected) exposure to unspecified communicable disease |
| Z22.3 | Carrier of other specified bacterial diseases |
| Z22.8 | Carrier of other infectious diseases |
| Z22.9 | Carrier of infectious disease, unspecified |
| Z76.0 | Encounter for issue of repeat prescription |

| **Supplementary Table 4. Demographics of all acute respiratory infection encounters** | | | | |
| --- | --- | --- | --- | --- |
| **Characteristic** | **Pre-intervention** | | **Post-intervention** | |
|  | **Control** | **Intervention** | **Control** | **Intervention** |
| ARI encounters (n=51,928) | 10,620 | 20,531 | 7,911 | 12,866 |
| Age (mean) | 50.8 | 42.3 | 49.8 | 42.7 |
| Female (%) | 61.1 | 58.7 | 61.2 | 57.9 |
| ARI prescribing rate (%) | 39.4 | 35.0 | 40.8 | 30.6 |
| ARI encounters where antibiotics are inappropriate (%)^a^ | 62.9 | 63.9 | 58.8 | 62.7 |
| ARI encounters where antibiotics are or may be appropriate (%)^a^ | 33.1 | 31.2 | 36.2 | 32.9 |
| ADI (mean) | 3.4 | 6.9 | 3.6 | 6.7 |
| Number of attending physicians | 41 | 116 | 45 | 114 |

ADI: Area depravation index^19,20^; ARI: Acute respiratory infection.

^a^ These rows do not sum to 100% in a given column because encounters with a secondary exclusion code listed in Supplementary Table 3 were excluded.
